# Supplementary material for: Vaccine discourse during the onset of the COVID-19 pandemic: Topical structure and source patterns informing efforts to combat vaccine hesitancy
Source: PLoS One. 2022 Jul 27;17(7):e0271394. doi: 10.1371/journal.pone.0271394 (PMC9328525; doi:10.1371/journal.pone.0271394)
Supplement: S1 Appendix — (DOCX) [file pone.0271394.s001.docx]

**S1 Appendix. keywords**

| Step | Goal | Keywords |
| --- | --- | --- |
| 1 | COVID-19 | 14dayquarantine, 2019ncov, 21dayslockdown, Avoidcrowds, "bars closed", Bayarealockdown, becool2asians, bioweapon, bioterrorism, breakcorona, californialockdown, californiashutdown,  californiaquarantine, calockdown, canceleverything, CDC, Carona,  "centers for disease control and prevention", chin*virus, "chinese virus",  "china virus", chinalie*, closenycpublicschools, codvid19, "codvid_19", Communistvirus, "community spread", convid19, conronaviruspandemic, corona*, "coron virus", Coronials, corono*, "corrona virus", Covd,  "covid-19", covid*, "covid-2019", curona*, disinfect, (("dont touch") NEAR/1 face), duringmy14dayquarantine, donttouch*face, epidemic, epitwitter, "essential service", "essential services", "face shield", "flatten the curve", "flattening the curve", Flu, flatten*thecurve, frontline*, "hand hygiene", hand*sanitizer, getmeppe, "health worker", Holdthevirus, Hubei,  Homeschool, Iamnotavirus, Hydroxychloroquine, iamnotcovid19, "incubation period", Inmyquarantinesurvivalkit, Koronavirus, Kungflu,  letsdefeatcovid19together, "li wenliang", lock*down, losangeleslockdown,  makechinapay, michiganshutdown, mask, mumbailockdown, n95, ncov*,  notdying4wallstreet, novelcorona*, ohiocoronavirus, pandemic,  outbreak, "panic shop", panic*bu, "personal protective equipment",  Plandemic, pmcares,Quarantine, Pneumonia, Ppe, quarentine*,  remoteworking, respirator, "restaurants closed",  "safer at home", Saferathome, "sars-cov-2", sarscov2, "self-isolation", Sendusbackhome, Sflockdown, Shamblesstayathome, Ppeshortage,  "sheltering in place", "shelter-in-place order", Shelteringinplace, shut*down, sideeffectsofquarantinelife, Sinophobia, "social distancing",  "social_distancing", socialdistanc*, "stay home", "stay at home", stay*home*, symptom, test, thankyouwarriors, toiletpaperpanic, "travel ban", "travel restriction", trump*demic, trumpliepeopledie, vegasshutdown, vaccine, ventilator, virus*, "wash hands",  Waragainstvirus, wash*hand*, wfh, whencoronavirusisover, whopaysforcovid, sanitize, work*from*home, wuhan,  "zhong nanshan", wearamask |
| 2 | Vaccine | “vaccine”, “vaccines”, “vaccination”, “vaccinated”, “vaccinate”, “vaccinating”, “covid-19 shot”, “covid19 shot”, “coronavirus shot”, “vax[a-z]^a^”, “anti-vax[a-z]^a^”, “biontech”, “Pfizer”, “mondera” |
