# Supplementary material for: Vaccine discourse during the onset of the COVID-19 pandemic: Topical structure and source patterns informing efforts to combat vaccine hesitancy
Source: PLoS One. 2022 Jul 27;17(7):e0271394. doi: 10.1371/journal.pone.0271394 (PMC9328525; doi:10.1371/journal.pone.0271394)
Supplement: S2 Appendix — (DOCX) [file pone.0271394.s002.docx]

**S2 Appendix. Codebook for positive and negative vaccine discourse on Twitter**

| Variable | Description | Example tweet |
| --- | --- | --- |
| Tweets with positive vaccine sentiment | If the tweet expresses positive attitudes or contain positive information about vaccination or comments from vaccine supporters  Note: if a tweet has discussion for both Covid-19 vaccine and other vaccine, code based on Covid-19 vaccine discourse. If a tweet only has discussion about other vaccine or general vaccination, code based on the discussion about other vaccine to infer attitude towards Covid-19 related vaccine^a^ | “Exactly they need to find a Vaccine just like they did the flu so that we will all be safe.”  “The vaccine is being developed in CT and according to them its 48 months off. I hope the FDA fast tracks the approval (48 mo includes the approval and production time)”  “Your baby’s immune system may be strong and can fight thousands of germs every day, but they can’t fight off all of them on their own. That’s where #vaccines come in. Join Jack and his parents as we explore #HowVaccinesWork. #immunizePA #VaccinesWork”  “You know that fear circling about #COVID19? The sense that quarantines and school cancellation are around the corner? Imagine that every summer. In 1952, 60,000 kids in the US were infected with polio and 3,145 died. There were 95 cases of polio worldwide last year. #VaccinesWork”  “Vaccines save millions of lives a year, but now the #COVID19 pandemic threatens children's access to routine immunizations for #polio, #measles, #HPV and more. \"The tragic reality is that children will die as a result\" @DrTedros #VaccinesWork #WorldImmunizationWeek2020 @WHO” |
| Tweets with negative vaccine sentiment | It the tweet expresses negative attitudes or contains negative information about vaccination, or comments from vaccine opponents | “Proof #vaccines are NOT tested properly before approval - WATCH NOW! Meaning... YOU ARE THE TEST SUBJECT! But your doctor won't tell you that... #LearnTheRisk #VaccineInjury”  “#DidYouKnow that #Measles mortality in the US was 2-in-a-Million BEFORE the #vaccination started? Check 1960 Vital Statistics of the US Vol II, Part A Table 1-M , page 1-27 Total MEASLES Death Rate: 0.2 per 100,000 #LearnTheRisk #WakeUpAmerica”  “US Government Pays Out Millions to Victims Killed or Paralyzed by #FluShot Of those 89 cases compensated, 77 were for injuries & 1 death caused by the #FluVaccine, making the #flu shot the most dangerous #vaccine in the US. #LearnTheRisk #VaccineInjury”  “It Isn't Just About #Autism, Folks! #Vaccine Induced Immune Overload & the Epidemic of #Chronic #Autoimmune Childhood Disease #LearnTheRisk #VaccineInjury #vaccines”  “#MMR #Vaccine Licensing Called Into Question Following ICAN's FOIA (#Freedom of #Information Act) Exposure of FDA Coverup MMR was licensed based on trials which in total had 800 participants & far more adverse reactions than acknowledged. #LearnTheRisk” |

^a^This rule is applied to both codes for positive and negative vaccine discourse.
