# Supplementary material for: Vaccine discourse during the onset of the COVID-19 pandemic: Topical structure and source patterns informing efforts to combat vaccine hesitancy
Source: PLoS One. 2022 Jul 27;17(7):e0271394. doi: 10.1371/journal.pone.0271394 (PMC9328525; doi:10.1371/journal.pone.0271394)
Supplement: S3 Appendix — (DOCX) [file pone.0271394.s003.docx]

**S3 Appendix. The fine-tuned BERT classification accuracy and Area Under Curve (AUC) (S3 Appendix Table) and the receiver operating characteristic curve (ROC) on the test set by positive and negative vaccine discourse (S1 Fig)**

|  | Accuracy | AUC |
| --- | --- | --- |
| Positive discourse | 0.71 | 0.79 |
| Negative discourse | 0.75 | 0.82 |
