# Supplementary material for: Vaccine discourse during the onset of the COVID-19 pandemic: Topical structure and source patterns informing efforts to combat vaccine hesitancy
Source: PLoS One. 2022 Jul 27;17(7):e0271394. doi: 10.1371/journal.pone.0271394 (PMC9328525; doi:10.1371/journal.pone.0271394)
Supplement: S4 Appendix — (DOCX) [file pone.0271394.s004.docx]

**S4 Appendix. The top 100 most mentioned accounts in positive and negative vaccine twitter discourses**

|  | Positive discourse | | Negative discourse | |
| --- | --- | --- | --- | --- |
|  | Account | Type | Account | Type |
| 1 | @realdonaldtrump^a^ | Political source | @realdonaldtrump^a^ | Political source |
| 2 | @cnn^a^ | Media/journalists | @billgates^a^ | - ^b^ |
| 3 | @potus^a^ | Political source | @potus^a^ | Political source |
| 4 | @cdcgov^a^ | Scientist/medical source | @who^a^ | Scientist/medical source |
| 5 | @who^a^ | Scientist/medical source | @mcfunny^a^ | Online influencer |
| 6 | @scottgottliebmd | Scientist/medical source | @doritmi^a^ | Scientist/medical source |
| 7 | @whitehouse^a^ | Political source | @whitehouse^a^ | Political source |
| 8 | @thehill^a^ | Media/journalists | @jkellyca^a^ | Suspended account |
| 9 | @nytimes^a^ | Media/journalists | @monstercoyliar^a^ | Online influencer |
| 10 | @billgates^a^ | - ^b^ | @janeeopie^a^ | Scientist/medical source |
| 11 | @msnbc | Media/journalists | @chrisjohnsonmd | Scientist/medical source |
| 12 | @joebiden^a^ | Political source | @cnn^a^ | Media/journalists |
| 13 | @washingtonpost | Media/journalists | @frankdelia7^a^ | Online influencer |
| 14 | @benshapiro | Media/journalists | @_mamadeb | Online influencer |
| 15 | @nbcnews | Media/journalists | @carlsmythe | Scientist/medical source |
| 16 | @foxnews^a^ | Media/journalists | @krebiozen | Online influencer |
| 17 | @gavi | Scientist/medical source | @plasticdoe | Online influencer |
| 18 | @abc | Media/journalists | @takethatcdc | Online influencer |
| 19 | @cnbc | Media/journalists | @ianfmusgrave | Scientist/medical source |
| 20 | @vp | Political source | @wendyorent | Scientist/medical source |
| 21 | @elonmusk | -^b^ | @bluelionblog | Online influencer |
| 22 | @berniesanders^a^ | Political source | @beckyjohnson222^a^ | Online influencer |
| 23 | @pfizer | Scientist/medical source | @stopvaxxedlies | Online influencer |
| 24 | @aslavitt | Scientist/medical source | @macbaird13 | Suspended account |
| 25 | @reuters | Media/journalists | @pjmoore1958 | Online influencer |
| 26 | @realcandaceo^a^ | Media/journalists | @joebiden^a^ | Political source |
| 27 | @speakerpelosi^a^ | Political source | @foxnews^a^ | Media/journalists |
| 28 | @mcfunny^a^ | Online influencer | @geoffschuler | Online influencer |
| 29 | @ap | Media/journalists | @cdcgov^a^ | Scientist/medical source |
| 30 | @wsj | Media/journalists | @thereal_truther | Online influencer |
| 31 | @doritmi^a^ | Scientist/medical source | @tiochango | Online influencer |
| 32 | @jnjnews | Scientist/medical source | @rosewind2007_ | Online influencer |
| 33 | @nygovcuomo | Political source | @realcandaceo^a^ | Media/journalists |
| 34 | @jacindaardern | Political source | @crabb_vicki | Online influencer |
| 35 | @nih | Scientist/medical source | @thehill^a^ | Media/journalists |
| 36 | @us_fda | Scientist/medical source | @cernovich | Online influencer |
| 37 | @jimcramer | Media/journalists | @med1cinewoman | Online influencer |
| 38 | @gavinnewsom | Political source | @speakerpelosi^a^ | Political source |
| 39 | @cbsnews | Media/journalists | @berniesanders^a^ | Political source |
| 40 | @senschumer | Political source | @nytimes^a^ | Media/journalists |
| 41 | @jkellyca^a^ | Suspended account | @smcwoof | Suspended account |
| 42 | @cnnbrk | Media/journalists | @fiski70 | Online influencer |
| 43 | @janeeopie^a^ | Scientist/medical source | @marthacurlee3 | Suspended account |
| 44 | @eugenegu | Scientist/medical source | @staci04907284 | Online influencer |
| 45 | @cepivaccines | Scientist/medical source | @phadingdark | Online influencer |
| 46 | @ monstercoyliar^a^ | Online influencer | @mtb_chum | Online influencer |
| 47 | @ beckyjohnson222^a^ | Online influencer | @tigerquinn7 | Online influencer |
| 48 | @ ingrahamangle | Online influencer | @mjonesnr | Online influencer |
| 49 | @npr | Media/journalists | @risettemd | Online influencer |
| 50 | @frankdelia7^a^ | Online influencer | @useemdumb | Suspended account |

^a^ The co-mentioned influential accounts.

^b^ These accounts do not fall into our five categories.
