# Supplementary figures and images for: Vaccine discourse during the onset of the COVID-19 pandemic: Topical structure and source patterns informing efforts to combat vaccine hesitancy
Source: PLoS One. 2022 Jul 27;17(7):e0271394. doi: 10.1371/journal.pone.0271394 (PMC9328525; doi:10.1371/journal.pone.0271394)

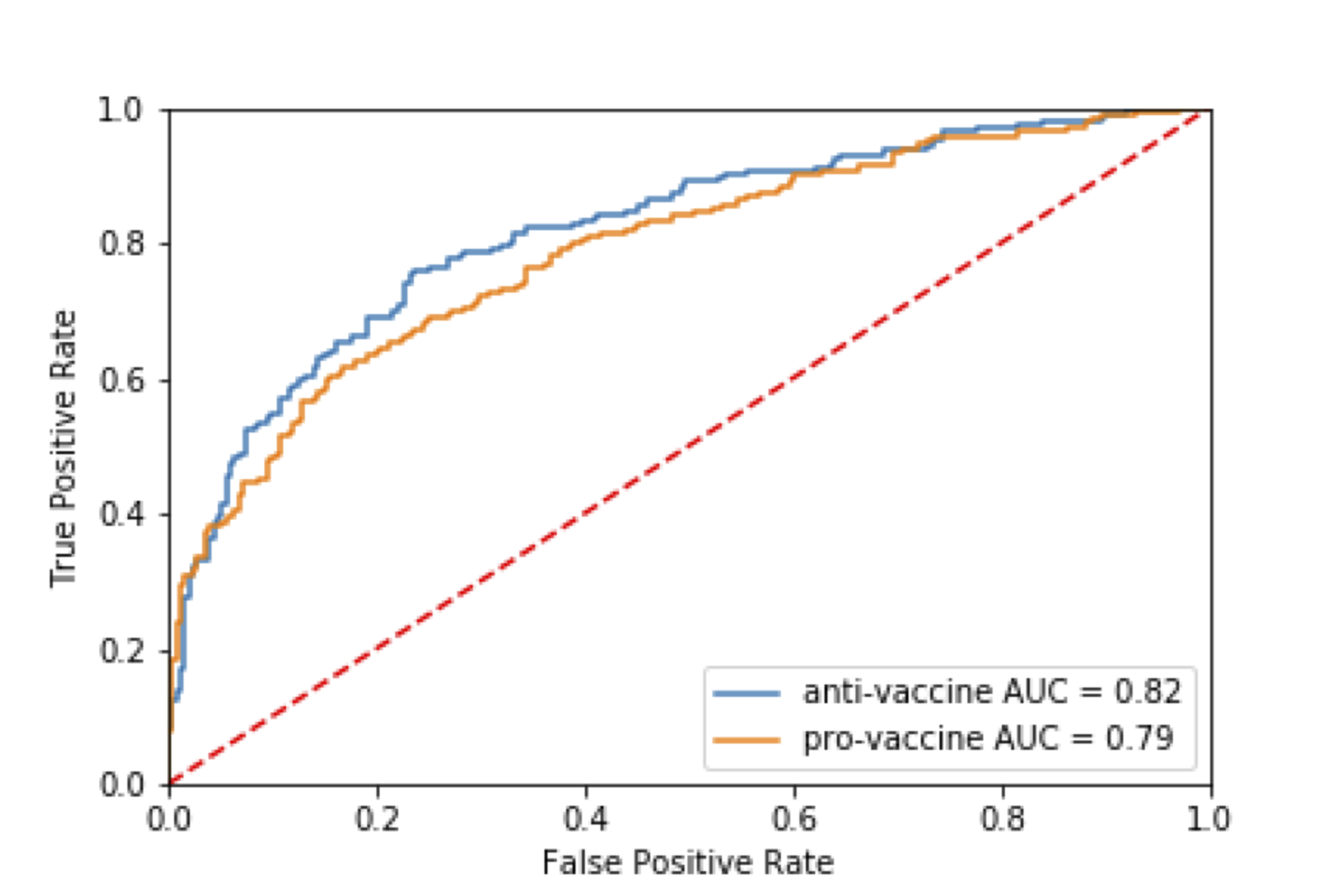

Supplement: S1 Fig — (TIF) [file pone.0271394.s005.tif]
