# Supplementary material for: Vaccine discourse during the onset of the COVID-19 pandemic: Topical structure and source patterns informing efforts to combat vaccine hesitancy
Source: PLoS One. 2022 Jul 27;17(7):e0271394. doi: 10.1371/journal.pone.0271394 (PMC9328525; doi:10.1371/journal.pone.0271394)
Supplement: Attachment — Submitted filename: Response letter_final_CLEAN.docx [file pone.0271394.s006.docx]

Dear Editor,

We appreciate the feedback from the reviewers and the opportunity to make further revisions on our manuscript for PLOS ONE. We have implemented all of the reviewers’ suggestions and believe that the manuscript is much strengthened as a result. Below, we list the feedback we received from the reviewers, and detail how we have responded to each concern in our revision of the manuscript.

Reviewer #1: This is a strong study that provides important insights into communication flows on Twitter during the early part of the COVID pandemic. It shows key differences between negative and positive sentiment through a comprehensive analysis (i.e., using multiple methods) of original tweets. The methods are sound, and findings support the conclusions. There also is real value here for public health communication.

My main recommendation is to tighten and revise the structure of the article in order to clearly draw out the core insights and to show why these methods are required to generate those insights.

Response: We appreciate the reviewer’s constructive comment. This important comment led us to revise our paper in a way that delivers our main points more effectively. To address this comment, in the introduction, we have now clarified our dual research goals – to identify patterns in the “content” of vaccine discourses on the one hand and to key “actors” with whom users engaging with the vaccine conversation interacted on the other. This revision is presented on page 6:

In the "Descriptive" section, it is somewhat difficult for the reader to understand how each part of this sequentially presented analysis builds to the article's findings and main argument. Why, for example, does this section of the article start by tracking the volume of tweets? What does this tell us about COVID communication flows that will be integral to the study's main argument?

Response: Thank you for this comment. We agree with the reviewer that each finding needs to be presented in a more coherent way and strung together to show the main line of our arguments. Thus, we have now clarified why we started by presenting the volume of tweets and how it would lead readers to the next findings. This revision is presented on page 10.

“We start by plotting the temporal dynamics of twitter volume during the course of our study time, with all types of tweets included, because the overall volume of tweets and how it evolved over time provides a big picture of our dataset before we delve into the examination of content of tweets.”

Each of these sections in the "Descriptive" section should be introduced with a clear statement of why the authors are choosing to, in order, analyze:

1) Volume of tweets
2) Prevalence of topics
3) Topic communities
4) Relative volume of topics over time
5) Distinct topical prevalence, positive vs. negative valence
6) Same as 5, but in relationship to hashtags
7) Mention network
8) Mention by account type

In other words, this is a richly detailed analysis that needs to be strung together in a more coherent fashion that, in each case along the way, builds and supports a clear argument based on the findings.

Response: We appreciate this constructive feedback. The reviewer’s comment truly led us to re-organize our findings in an effective way. We have now made two changes in the results section. First, we have broken down the results section into two major sections (3.1 Content and 3.2 Actors). Second, under each section, we have assigned six subsections for 3.1 Content (3.1.1 Volume of tweet, to 3.1.6 Distinct topical prevalence in relation to hashtags) and two subsections for 3.2 Actors (3.2.1 Mention network and 3.2.2 Mention by account type). This revision allows our findings to be more clearly presented. We thank the reviewer for this very constructive comment.

More generally, the findings need to be more closely stitched together with the very clear enumeration of issues of 1) content, and 2) actors on pp. 27-28. Revising the article to support, step-by-step, this dual focus on content and actors will help bring more coherence to the methods and findings.

Response: We appreciate this feedback. As stated in the previous responses, we have now clarified our dual purposes for this paper and consistently emphasized them throughout the manuscript in the introduction, methods, results, and discussion.

It also would be nice to see a concise, brief summary of the study's three principal takeaways in the first paragraph of the Discussion. This could be one or two sentences following the mention of topics and actors, which then would lead into the more detailed elaborations of each individual finding below.

Response: Thank you for this comment. We agree with the reviewer that it would be helpful to briefly summarize our study’s principal takeaways in the first paragraph of the discussion. Thus, we have now included a summary before delving into the detailed discussion of each finding:

“Our study examined a) *content* difference in positive versus negative tweets surrounding the COVID-19 vaccine, as well as b) prominent *actors* frequently mentioned by the two camps. The primary findings were summarized as follows: While positive vaccine discussions demonstrated a wider range of perspectives informed by research and medical professionals, negative vaccine conversation was dominated by a narrower set of narratives, such as monetary motivations behind mandatory vaccines, concerns over side effects, as well as conspiratorial beliefs. Furthermore, while positive vaccine discourse interacted with verified agents, negative vaccine discourse tended to mention online influencers and suspended accounts, suggesting possible pathways through which misinformation was spreading.”

Also helpful for maximizing the article's impact would be noting present public health communication strategies designed to counter vaccine hesitancy. These could be added to the introduction's literature review, and they would help clarify what is new about the author's conclusions and recommendations for improving these strategies and developing new strategies.

Response: Thank you for the important comment. We have now created a separate paragraph that notes the present public health communication strategies and shows how our study can provide practical implications based on the current approach. The paragraph at the end of the introduction on page 6 reads:

“By doing so, our study provides important insights and practical guides for public health communicators, which, to date, predominantly focus on disseminating vaccination guidelines or schedules, rather than proactively responding to vaccine hesitancy. Thus, we suggest public health communication professionals should broaden their messages to address specific concerns as revealed in public conversations on social media, as well as leverage a diversity of actors who have been centrally situated in the vaccine-hesitant communities.”

A few other issues:

The authors use the terms "themes," "thematic patterns," and "topics" interchangeably. I suggest sticking to a single term, such as "topic," unless there is a distinction between themes and topics that is important to the study. If so, this distinction needs to be clarified.

Response: Thank you for the important comment. Indeed, we should stick to a single term, which describes our measurement accurately. We have now used “topics” consistently and removed all the “themes.” We have also revised our title accordingly. We appreciate the reviewer’s comment.

The methods arise in mid-paragraph on p. 5. All references to methods to be contained in the methods section.

Response: We thank the reviewer for this comment. We have now removed any mention about methods from the introduction. We have specifically removed our statement about the five categorizations of account mentions from this section and moved it to the method section on page 10. We appreciate the reviewers’ comment.

Something is missing in the first sentence on p. 17. "To understand how the discourses" what?

Response: We have corrected this typo.

In Footnote 3 on p. 20, please clarify that @realdonaldtrump is former President Trump's Twitter account. As it now reads, "his" is a pronoun without a first reference.

Response: Thanks for this comment. We have now clarified that this account is the former President Trump’s in the footnote 3 on page 21.

p. 11: Figure 1 has a typo in the title: "Volumn"

Response: Thanks for the attention to detail. We have corrected this typo.

p. 23: "Principle findings" should be "Principal findings"

Response: We have corrected this typo.

p. 24 and p. 29: the term "anti-vaxxers" is used here, without definition, though the term implies that all of these social media users were actively opposed to vaccinations. However, we know that there is a range of negative vaccine sentiments, from hesitancy and doubt, to criticism and outright opposition. Accordingly, the literature in this area tends to invoke a spectrum of vaccine confidence (see Larson, 2020; MacDonald & the SAGE Working Group on Vaccine Hesitancy, 2015), and the authors open the article by referring to this spectrum. It then is confusing why the term "anti-vaxxers" appears at this point in the article. Please clarify or revise the terminology to align with the article's introduction.

Response: We appreciate this important comment. We agree that the appearance of the term, “anti-vaxxers” in this stage of the paper might confuse the readers for several reasons mentioned by the reviewer. We also agree that it is inappropriate to mention this term without definition, since it may lead readers to overlook the dynamic of negative attitudes toward vaccines. We have now removed this term and replaced it with “users with negative attitude toward the COVID-19 vaccine.” We have also made sure that the expression is consistently used throughout the manuscript (e.g., positive/negative attitude toward COVID-19 vaccine) to properly reflect the nuanced nature of this spectrum in negative attitudes, as the reviewer well-pointed out. We thank the reviewer for this comment.

---

Reviewer #2: Overall

The general area of research is topical. Overall I don't find this paper compelling, partly due to the methods, and partly due to the execution. I am not an expert in STM, so I cannot comment on that. If they could do the paper without machine classification, I think it would be improved, as I don't think these methods are successful on Twitter data--based on their results and my personal experiences.

Major comments

I have done extensive work using machine learning and Twitter, and have never published it because the results are always disappointing. My experience is that 140 characters is not sufficient for proper classification of most Twitter content. The authors used a machine learning method that has, in my view, high error, reporting only 71% and 75% accuracy. The ROC curves in vaccine 3 confirms fairly high combined error tradeoff between sensitivity and specificity. The fact that that 20-30% of the content is coded wrong should cause the authors to be very cautious in the interpretation of their results, and I think presents a serious challenge to publishing this paper. The use of regression models in analyzing an outcome with large measurement error (Table 2) is especially problematic.

Response: Thanks for the reviewer’s important comment. We resonate with the reviewer’s concerns that conducting machine learning, and especially with Twitter data, is not easy. As with other methodologies, machine learning-based approaches certainly have their limitations, as we acknowledged in the manuscript. Nevertheless, machine-learning approach provides a scalable solution to text classification tasks with consistent criteria, adding important insights to our existing knowledge on the topic. There are several advantages of our current approach. First, compared to other unsupervised textual analysis techniques, machine-learning allows tracking discourses that are theoretically important or comparing discourses between groups on the same criteria (e.g., discourses about anti-COVID-19 vaccine). Similarly, when compared with other research methods such as survey, machine-learning approach helps to detect naturally occurring public expression of certain topic or sentiment unobtrusively.

We also want to emphasize that there are a significant number of published papers that used machine-learning method with Twitter data (Below we listed a few examples).

Please also let us note that the classification solutions were validated with several additional procedures, including manual coding and structural topic modeling. The accuracy level (71% and 75%) also seems acceptable in our downstream application task, as indicated in other published work (e.g., 74.1% in Zhu et al.,2020).

Lastly, as for the regression models in Table 2, please let us clarify that the numbers are coefficients in the regression model, which is not an index of measurement error.

Allen, C., Tsou, M. H., Aslam, A., Nagel, A., & Gawron, J. M. (2016). Applying GIS and machine learning methods to Twitter data for multiscale surveillance of influenza. PloS one, 11(7), e0157734.

SteelFisher, G. K., Blendon, R. J., & Caporello, H. (2021). An uncertain public—encouraging acceptance of Covid-19 vaccines. New England Journal of Medicine, 384(16), 1483-1487.

Xue, H., Bai, Y., Hu, H., & Liang, H. (2019). Regional level influenza study based on Twitter and machine learning method. PloS one, 14(4), e0215600.

Zhu, J. M., Sarker, A., Gollust, S., Merchant, R., & Grande, D. (2020). Characteristics of Twitter use by state medicaid programs in the United States: machine learning approach. Journal of medical Internet research, 22(8), e18401.

I don't know much about structural topic modelling, but these kinds of approaches are more effective when there is more text available. Again, Twitter data presents a challenge here; gathering a meaning from small quantities of text is hard. However, since i am not an expert in this method, I cannot comment further.

Response: Thank you for this comment. We observe the research communities that are still in the process of advancing algorithms for detecting topics in Twitter data. Nevertheless, structural topic modeling and topic modeling, while not ideal as with any other method, is an established approach for analyzing Tweets. Below are some relevant publications that use structural topic modeling for twitter data analysis.

Surian, D., Nguyen, D. Q., Kennedy, G., Johnson, M., Coiera, E., & Dunn, A. G. (2016). Characterizing Twitter discussions about HPV vaccines using topic modeling and community detection. Journal of medical Internet research, 18(8), e6045.

Mishler, A., Crabb, E. S., Paletz, S., Hefright, B., & Golonka, E. (2015, August). Using structural topic modeling to detect events and cluster Twitter users in the Ukrainian crisis. In International Conference on Human-Computer Interaction (pp. 639-644). Springer, Cham.

Finally, I don't see the practical implications of the paper. The authors claim that "our results highlight the type of content that needs to be addressed to improve vaccine hesitancy" but offer no actual evidence of this. This is a desscriptive paper that does not drill into what imapats vaccine hesitancy. Moreover, changes in attitudes towards covid-19 vaccines render some of these findings somewhat obsolete; what concerns people today (in late 2021) may be different from what concerned them early in vaccine uptake.

Response: Thank you for the important comment. As the reviewer pointed out, this paper is not providing direct evidence that certain content can reduce vaccine hesitancy. However, our findings do identify specific areas of concerns that can serve as leverage points to create more effective and tailored messages. We believe this is where our practical implications are.

We also agree with reviewer’s comment that our results from the early phase of the pandemic might be different from the results from the current phase. Our goal of this paper, however, is to highlight vaccine discourses particularly occurring in the early stage of the pandemic. It is reasonable to expect that, when the pandemic began or when the COVID-19 vaccine first became available, the fear or levels of uncertainty about COVID-19 vaccine was unprecedented. It was also the time where all kinds of mis- and disinformation have begun to gain steam. Thus, we intended to detect topical themes, topic prevalence, and network communities regarding pro-vaccine and anti-vaccine discourses in this particular stage. On the other hand, it was also a critical point where health professionals can intervene to shape public perception and spread science-based messages. As such, it is our belief that exploring the topics and prominent actors in this critical period has values in and outside the Covid-19 context. Per the reviewers’ comment, we have now clarified our practical implication in the introduction on page 6:

“By doing so, our study provides important insights and practical guides for public health communicators, which, to date, predominantly focus on disseminating vaccination guidelines or schedules, rather than proactively responding to vaccine hesitancy. Thus, we suggest public health communication professionals should broaden their messages to address specific concerns as revealed in public conversations on social media, as well as leverage a diversity of actors who have been centrally situated in the vaccine-hesitant communities.”

Minor comments

Page 4 "social media content does not undergo an editorial processes or scientific vetting". This statement is not true. Twitter, Facebook and YouTube implemented editorial controls specific to Covid and vaccination in 2020.

Response: Thank you for the comment. Here, we wanted to emphasize that the social media contents, as expressions from individual users, do not generally go through editorial processes before entering the public sphere. Different from other types of contents such as news articles, social media contents represent individuals’ opinions in a more direct way, and with fewer constraints. As the reviewer pointed out, some platforms implemented editorial controls such as rules to curb the spread of misinformation. Nevertheless, social media contents still reflect individuals’ expression primarily because the contents are only subject to the editorial control (i.e., removal) after, not prior to production. More importantly, a variety of opinions still have presence after the editorial control. For example, though Twitter has policies in place to remove tweets containing false claims about COVID-19, there is a fine line to draw between "undisputed" scientific facts and freedom of expression. As a result, strong sentiment or opinions that contradict official recommendations or against the best available scientific evidence were often left to thrive online. Due to the fundamental difference in epistemic production, social media contents like tweets are not bound by common newsroom practice, leading to an "unedited" public sphere (Bimber & Gil de Zúñiga, 2020, p.700).

Bimber, B., & Gil de Zúñiga, H. (2020). The unedited public sphere. *New Media & Society*, *22*(4), 700-715.

We also referred to Twitter’s COVID-19 misleading information policy (<https://help.twitter.com/en/rules-and-policies/medical-misinformation-policy>).

Thus, per the reviewer’s comment, we have now clarified our sentences:

“In contrast to traditional news media or scholarly outlets, social media content does not undergo an editorial processes or scientific vetting unless violate certain rules of a platform, thereby allowing users to voice their opinions on their own terms in most cases (p.4).

Page 6. Is the use of Synthesio and web scraping of Twitter legal? Is the use of this tool covered under fair use legislation/practice? Do we know that Synthesesio actually generates true and unbiased samples? Was this service paid for by the authors? Details here are important for the reader for a number of reasons.

Response: Thank you for the comment. Synthesio is a widely used, paid platform to obtain social media data. It is a legal third-party platform and we were authorized to use the platform by paying for it. It is a random sample. While it is possible that data quality is compromised by restrictions such as caps on the maximum return or the total daily queries allowed to run, our data is not obviously biased since Synthesio uses random sampling. Please refer to this website for more information about Synthesio’s dashboard for social media: <https://www.synthesio.com/products/social-listening/>.

We have clarified that it is a random sample on page 6:

“We first scraped Twitter data through Synthesio, a social media monitoring tool to randomly sample 1% of public tweets using COVID-19 related keywords.”

We appreciate all the constructive comments, and we believe we have strengthened our paper considerably by addressing them.
