# Supplementary material for: Vaccine discourse during the onset of the COVID-19 pandemic: Topical structure and source patterns informing efforts to combat vaccine hesitancy
Source: PLoS One. 2022 Jul 27;17(7):e0271394. doi: 10.1371/journal.pone.0271394 (PMC9328525; doi:10.1371/journal.pone.0271394)
Supplement: Attachment — Submitted filename: Response letter.docx [file pone.0271394.s007.docx]

Dear Editor,

We appreciate the feedback we received from the reviewers and the opportunity to make further revisions on our manuscript for PLOS ONE. We have implemented all of the reviewers’ suggestions and believe that the manuscript is much strengthened as a result. Thank you. Below, we list the reviewers’ feedback, and detail how we have responded to each concern in our revision of the manuscript.

Reviewer #1: The authors have improved the readability of the article by providing effective subheadings, by more strongly emphasizing the two-fold nature of the analysis (content/actors), and by including additional cites and cautions regarding the methods. I do think that these methods are appropriate for Twitter analysis, though they must be presented with caution, as the authors do.

My only remaining question concerns the authors' recommendation of paying for the promotion of content among vaccination hesitant Twitter users, as I am unclear of ethics considerations or other potential concerns surrounding such an intervention. Are there cites that could be included to support this recommendation or at least to point out relevant debates concerning it?

Response: We appreciate the reviewer’s important comment. We originally included this practical suggestion by observing the increasing efforts to reduce misleading information policies, which are very creative and proactive. This recommendation, however, should be suggested with caution because it could produce potential ethical issues, as the reviewer pointed out. Thus, after careful consideration, we have removed this recommendation on page 25. We are very grateful to the reviewer for the attention to detail.

Reviewer #3: Authors explore public discourse about covid 19 via random sample of twitter and analyzed using Structural Topic Modeling and Network Analysis.

I am satisfied by most of responses provided by the authors on the reviewes. However, I would argue that authors still need to highlight/defend their selection of methods. Since this same study can be conducted using multiple methods in unsupervised learning, and there is much research available for this as well, it is important that authors explain their strategy of selecting this method. I,e sentiment could also capture using other NLP methods, authors may have a reason to use the ML model instead.

Response: Thank you for the constructive comment. We agree with the reviewer that a justification on our selection of supervised machine learning will further strengthen our manuscript. Thus, we have added the following paragraph on pages 7-8:

“Specifically, we used a transformer-based supervised machine-learning method, which allows labeling a tweet as expressing positive or negative sentiment regarding COVID-19 vaccines, and in turn, classifying the rest of the tweets based on the labeled samples using the Bidirectional Encoder Representations from Transformers (BERT) machine learning algorithm. There are several advantages with our current approach. Compared to other unsupervised textual analysis techniques, supervised machine-learning allows tracking discourses that are theoretically important or comparing discourses between groups on the same criteria. In our case, our supervised machine learning approach provides a computationally efficient way to classify tweets based on target-specific sentiments (i.e., sentiment toward vaccines), rather than more generic tasks of sentiment detection. By contrast, unsupervised machine learning is to learn the inherent structure of the data without pre-provided labels [40,41]. Since the performance of the unsupervised learning-based sentiment analysis methods heavily relies on the pre-generated lexicon and corpus, if the sentiment/emotion relevant words do not exist in the lexicon or corpus, the classification accuracy will be low [40,41]. Given that COVID-19 and its vaccines involve high levels of uncertainty and novelty, as well as its nature of constantly evolving, we believe that a supervised machine-learning approach would be appropriate to provide a scalable solution to text classification tasks with pre-defined criteria.”

[40] Hu, T., Wang, S., Luo, W., Zhang, M., Huang, X., Yan, Y., ... & Li, Z. (2021). Revealing Public Opinion Towards COVID-19 Vaccines With Twitter Data in the United States: Spatiotemporal Perspective. Journal of Medical Internet Research, 23(9), e30854.

[41] Wang, S., Huang, X., Hu, T., Zhang, M., Li, Z., Ning, H., ... & Li, X. (2022). The times, they are a-changin’: tracking shifts in mental health signals from early phase to later phase of the COVID-19 pandemic in Australia. BMJ Global Health, 7(1), e007081.

At the same time, it would be helpful to know how did you ended up 2 coders, and were they experienced researchers or external from this study – this will help up to navigate the validity of the codes.

Response: Thank you for the comment. We had exerted extra cautions when inviting two coders to increase validity of codes as well as reduce any bias. We had two criteria. First, we intentionally excluded experienced researchers who conceptualized and designed this study from the coder pool to prevent any potential bias in codes. Second, familiarity with the primary concept and basic background or experience in content coding are of importance. Based on these criteria, two graduate assistants familiar with the domain subject and content analysis methodology, but not directly involved in designing this research, served as the coders for this study. We have now clarified this information on page 8.

I am not sure how did authors classify the mention network into several subjects – were these done by authors? 1 author? inductively or deductively?

Response: We appreciate this comment. We created classifications of the mention network both inductively and deductively. On one hand, we created primary categories (e.g., scientist/medical source, media/journalists, political source) based on the literature that emphasizes the degree to which the source provides access to medical expertise (inductive process). This process was designed as iterative where all members in the research team compared notes and validated with actual sample tweets until all major categories important to this study were captured. On the other hand, we identified the top 100 twitter accounts that have been mentioned by other users in our sample (Appendix 4) and manually assigned each a category label based on the literature. In this process, we recognized that there were a substantial number of accounts that didn’t fall into the primary categories; thus, two new categories– online influencer and suspended account– were added (deductive process).” It also came to our attention that one account may be classified into more than one category (e.g., a certified doctor who is also an online influencer); thus, we created a priority to classify accounts (deductive process). All of these procedures were completed based on discussion between authors. The following reference was added:

[39] Hwang, J., & Shah, D. V. (2019). Health information sources, perceived vaccination benefits, and maintenance of childhood vaccination schedules. Health Communication, 34(11), 1279-1288.

We have now clarified this information on page 11. We are grateful for the reviewer to provide us with an opportunity to clarify our procedures in the manuscript.

Topical associate represents in the figure 3 needs bit more details – how the association was derived between topics. May be one example to reflect the association would be more helpful to the reader.

Response: Thank you for the comment. We agree with the reviewer that additional explanation on how to interpret the association using edges is needed. Thus, we added the following paragraph on pages 16-17.

“The association between two topics (i.e., nodes) is reflected by the thickness of the undirected line connecting them (i.e., edge). The edges are weighted, calculated based on cosine similarity, with the weight indicating topic co-occurrence in documents. Specifically, the higher the weight, the more likely that two topics are discussed within a given document. For example, vaccine production is more likely to be talked about alongside other coping strategies, yet it has a low cosine similarity value with the topic of conspiracy [28]."

Near the figure 4 where authors present the temporal volume, authors state “…While some topics received more sustained overtime attention (e.g., Topic 6 – Conspiracy Theory), others showed a higher degree of fluctuation in Twitter volume (e.g., Topic 8 – Vaccine Development).” – It is not possible to derive this statement from figure 4, it should be explained using figure 3 and it need to bring the connection to figure 4.

Response: Thank you for the comment. We have now added a note in Figure 4 to add clarity of its interpretation as well as to bring connection.

Figure 6 is not words its #tags right, ( words can be misunderstood as words in the topic,but this not the case right? -> “Words featuring most prominently in positive versus negative vaccine discourses”.. Besides word cloud seems not much providing the context to what authors trying to explain in this. So its more taking space without much value.

Response: We appreciate your attention to detail. We originally included results about distinct topical prevalence in relation to hashtags to further clarify the difference between positive and negative vaccine discourses. However, we also feel that these results do not provide much novel information; thus, we have decided to remove these results.

Overall, I also felt the need of the coherent story and the key findings in the discussions and authors somewhat have addressed that, but this is still could be improved.

Overall, I enjoyed the implication part the most, which is the essence of this this contribution, and with that I think this paper has a substantial value to the journal.

We appreciate all the constructive comments. We have gone through the discussion to present our key findings more effectively. We have also tried our best to improve coherency in this section. We believe we have strengthened our paper considerably by addressing the reviewers’ comments. Thank you.
