# Supplementary material for: Vaccine discourse during the onset of the COVID-19 pandemic: Topical structure and source patterns informing efforts to combat vaccine hesitancy
Source: PLoS One. 2022 Jul 27;17(7):e0271394. doi: 10.1371/journal.pone.0271394 (PMC9328525; doi:10.1371/journal.pone.0271394)
Supplement: Attachment — Submitted filename: Response letter.docx [file pone.0271394.s008.docx]

Dear Editor,

We appreciate the feedback we received from the reviewers and the opportunity to make further revisions on our manuscript for PLOS ONE. We have implemented all of the reviewers’ suggestions and believe that the manuscript is much strengthened as a result. Thank you. Below, we list the reviewers’ feedback, and detail how we have responded to each concern in our revision of the manuscript.

Reviewer #1: This article is much improved and, in my view, needs only a couple minor revisions:

On p. 10: Should "Sandford" be "Stanford"?

Response: We appreciate your attention to detail. We have corrected this typo and proofread the manuscript again. Once again, we thank the reviewer for the comment.

p. 25: The authors indicated in their responses to reviewer comments that they had removed references to paying for promotion of content, yet this sentence remains in the article: "Again, paid sponsorship of influencers accounts may prove to be an effective intervention strategy." Do they still intend to include such recommendations?

Response: We appreciate the reviewer’s important comment. This recommendation, along with references to paying for promotion of content, should be removed, as it could produce potential ethical issues. We apologize for not getting it right in advance. We have now removed this sentence and are very grateful to the reviewer for the comment.

Reviewer #3: I thank the authos for addressing my comments in the manuscript and also explaining in the response letter. New changes appearing much improved and clear decription of the conduct and results and the story behind the results. I think the insights will bring much value to the readers.

Response: We appreciate all the constructive comments in the previous round. We believe we have strengthened our paper considerably by addressing the reviewers’ comments. Thank you.
